# Bile acid pathways in Caprinae gut microbiota: adaptive shifts in microbial metabolism and community structure

**DOI:** 10.3389/fmicb.2025.1648896

**Published:** 2025-09-12

**Authors:** Kai-Meng Shang, Rui Liu, Hong-Bo Ni, He Ma, Jin-Wen Su, Hai-Long Yu, Li Guo, Bei-Ni Chen, Xiao-Xuan Zhang, Xing Yang

**Affiliations:** ^1^Integrated Laboratory of Pathogenic Biology, College of Preclinical Medicine, Dali University, Dali, China; ^2^College of Life Sciences, Changchun Sci-Tech University, Shuangyang, Jilin, China; ^3^College of Veterinary Medicine, Qingdao Agricultural University, Qingdao, Shandong, China; ^4^College of Animal Science and Technology, Jilin Agricultural Science and Technology University, Jilin, China

**Keywords:** gut microbiota, microbial functional profiling, metagenomic, bile acid metabolism, Caprinae

## Abstract

**Introduction:**

The gut microbiota plays a central role in host metabolism and immunity, in part through bile acid (BA) biotransformation. In Caprinae animals such as goats and sheep, this process is critical for nutrient absorption, immune regulation, and intestinal homeostasis, yet the microbial taxa and functional pathways involved remain poorly characterized.

**Methods:**

By leveraging 7,530 high-quality non-redundant metagenome-assembled genomes (MAGs) from Caprinae gut microbiomes, this study systematically characterized microbial diversity, taxonomic composition, and bile acid (BA)-related metabolic pathways through genome annotation, phylogenetic inference, and statistical analyses.

**Result:**

The results revealed a diverse gut microbiota across 28 phyla, with *Bacillota*_A being the most dominant. A significant number of genes (8,290) from 5,217 genomes were identified to be involved in BA transformation pathways, including deconjugation, oxidation, and dehydroxylation. Bacteria from the *Bacillota*_A phylum were the primary carriers of BA-related genes. Among the MAGs, 1,845 encoded bile salt hydrolase (BSH), an enzyme crucial for the initial step of BA metabolism. Comparative analysis with human and pig gut microbiota highlighted a distinct BA metabolic profile in Caprinae animals, characterized by a higher proportion of BSH-related genes. Functional profiling of BSH-carrying MAGs within the genus *Alistipes* revealed significant differences in carbohydrate-active enzymes (CAZymes), indicating distinct metabolic repertoires that may reflect divergent ecological roles in the intestinal environment. Microbial taxonomic composition and bile acid (BA)-metabolizing potential varied markedly across the ten intestinal segments of *Ovis aries*, with the colon, cecum, and rectum showing the highest microbial diversity and functional gene abundance. Key BA-transforming enzymes (BSH, 7α-HSDH, and baiB) were widely distributed, with particularly high abundances in the jejunum and ileum, indicating region-specific specialization in BA metabolism.

**Discussion:**

This study provides new insights into the ecological and metabolic functions of gut microbiota in Caprinae animals, emphasizing the unique BA metabolic profiles and the functional potential of BSH-carrying MAGs, which have broader implications for understanding host-microbiota interactions in health and disease.

## 1 Introduction

Microbes are ubiquitous in nature and inhabit virtually all potential habitats (Alnahhas et al., 2020). Among these, a considerable diversity of microorganisms resides within the mammalian gastrointestinal tract, forming a highly intricate and dynamic microecological system (Heintz-Buschart and Wilmes, 2018). Increasing evidence suggests that host physiological processes-including metabolic pathways, immune responses, and energy homeostasis-can be modulated through complex interactions between the host and its gut microbiota.

As the largest symbiotic ecosystem within the host, the gut microbiota plays a critical role in maintaining intestinal homeostasis (Shang et al., 2025; Cheng et al., 2024). These microbes interact with the host to produce various metabolites, such as bile acids (BAs), which contribute not only to the balance of the microbial community but also to the preservation of mucosal integrity (Cai et al., 2022b). BAs are potent metabolic and immune signaling molecules synthesized from cholesterol in the liver and secreted into the intestine, where they undergo extensive biotransformation by the gut microbiota (Ridlon and Gaskins, 2024). The combined enzymatic activities of the host and intestinal microbes determine the composition of the BAs pool, resulting in considerable inter-individual variation that is partially driven by microbial diversity (Cai et al., 2022a).

Caprinae, which includes species such as goats (*Capra hircus*) and sheep (*Ovis aries*), are of significant agricultural importance worldwide due to their contributions to meat, milk, fiber, and ecological sustainability (Guerra et al., 2022). In these animals, the gut microbiota is believed to be essential not only for the degradation of plant-derived polysaccharides but also for BA metabolism-a process that influences lipid absorption, immune regulation, and overall intestinal equilibrium (Zhang et al., 2024). Key microbial enzymes such as bile salt hydrolase (BSH), 7α-dehydroxylase, and hydroxysteroid dehydrogenases (HSDHs) contribute to the deconjugation and transformation of primary BAs into diverse secondary BAs (Li and Chiang, 2023).

In this study, a genome-resolved analysis of the gut microbiota in Caprinae animals was conducted, with a particular focus on microbial taxa associated with BA metabolism and their functional capabilities. By leveraging a high-quality metagenomic dataset, we systematically examined microbial diversity, taxonomic composition, and BA-related metabolic pathways. These findings offer novel insights into the ecological roles and metabolic functions of gut microbiota in Caprinae, and provide a foundation for broader understanding of host-microbiota interactions in both health and disease contexts.

## 2 Materials and methods

### 2.1 Data collection and preprocessing of genomes and gene prediction

This study generated a dataset of 63,126 gut microbiome MAGs from Caprinae animals by assembling and binning publicly available metagenomic datasets. Detailed sample information, including host species, sample number, and BioProject accession numbers, is provided in Supplementary Table 1. Genome quality was assessed using CheckM2 (Chklovski et al., 2023) (v1.0.1) to evaluate completeness and contamination. To ensure high data integrity, more stringent quality thresholds were applied than those used by Lin et al. (2023), retaining only MAGs with ≥ 80% completeness and ≤ 5% contamination for downstream analyses. Deduplication was conducted using dRep (Olm et al., 2017) (v3.4.3) with the parameters: −pa 0.9, −sa 0.99, −nc 0.30. The resulting non-redundant, high-quality MAGs were taxonomically classified using the classify_wf workflow implemented in GTDB-Tk (Chaumeil et al., 2022) (v2.3.2). Open reading frames (ORFs) for these genomes were predicted using Prodigal (Hyatt et al., 2010) (v2.6.3). To infer phylogenetic relationships among the MAGs, a maximum likelihood tree was constructed using PhyloPhlAn (Asnicar et al., 2020) (v3.0.67), and visualization was performed using the iTOL (Letunic and Bork, 2021) (v6.9.1). To quantify the abundance of strain-level genomes (MAGs), samples from a previous study were collected from various regions of the gastrointestinal tract (GIT) (Jiang et al., 2022; Supplementary Table 2). These reads were aligned to the genome using Bowtie2 (Langmead and Salzberg, 2012) (v2.5.0) with default parameters. The mapped read counts were then normalized to transcripts per kilobase million (TPM).

### 2.2 Functional gene annotation

Functional annotations were performed using DIAMOND (Buchfink et al., 2015) (v2.1.8.162) to search the Kyoto Encyclopedia of Genes and Genomes (KEGG) database. KEGG orthologs (KOs) were filtered to focus on those related to secondary bile acid biosynthesis (map00121). Based on the available data for genes encoding the target KOs, their genomic origins were identified, and the copy numbers of these genes were calculated within individual MAGs. To further explore the functional capabilities, protein-coding genes were annotated by aligning them against the Carbohydrate-Active enZymes (CAZymes) database (Cantarel et al., 2009) using DIAMOND with the parameters “–min-score 60 –query-cover 50.” For each open reading frame (ORF), the alignment with the highest bit score was selected as the representative hit for both taxonomic and functional annotation.

### 2.3 Statistical analyses and visualization

Data analyses were conducted using R version 4.2.2. Taxonomic and functional gene abundance data were used to calculate the Richness and Shannon indices. β-diversity was assessed through Principal Coordinate Analysis (PCoA) based on Bray-Curtis distance, and group differences were evaluated using permutational multivariate analysis of variance (PERMANOVA). The Wilcoxon rank-sum test was applied to assess the significance of differences in diversity indices, taxonomic units, and functional gene feature abundances across groups. The “ggsankey” package (v0.0.9) was used to visualize the sankey plot, while “ggtern” package (v3.5.0) was used to visualize the ternary plot. All other visualizations were generated using the “ggplot2” package (v4.2.3).

## 3 Results

### 3.1 Collection of genomes from the intestines of Caprinae animals

**FIGURE 1:**
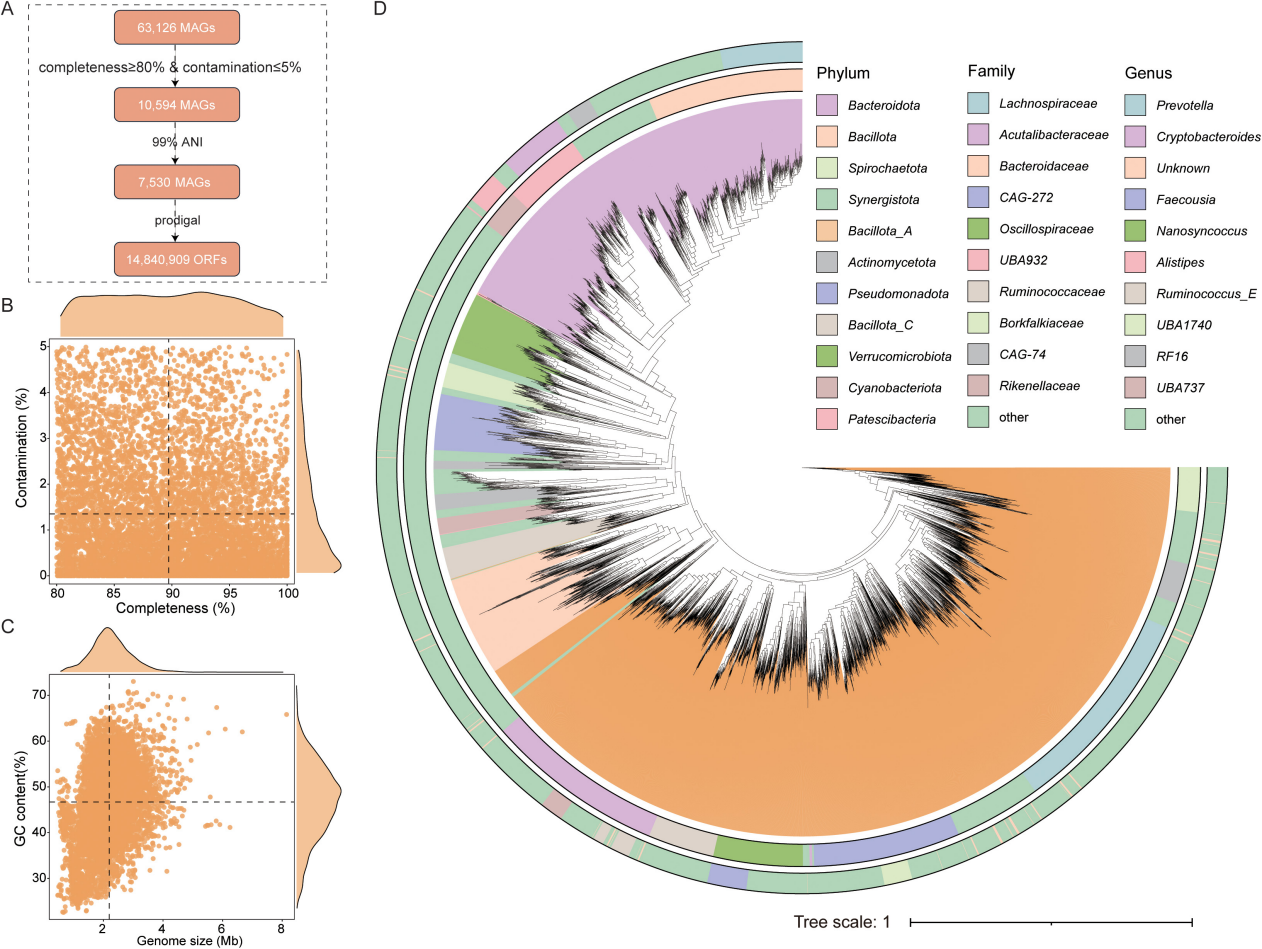
Genomic information collected for analysis. **(A)** Workflow for identifying and processing the genomes in this study, including quality assessment and redundancy removal. **(B,C)** Genomic statistics of the 7,530 metagenome-assembled genomes (MAGs), including their completeness, contamination, size, and GC content. **(D)** Taxonomic classification of 7,530 MAGs across hierarchical levels. Rectangles represent different taxonomic ranks, with their lengths proportional to the number of genomes assigned to each level.

To obtain more comprehensive genomic data on the gut microbiota of Caprinae animals, this study collected a total of 63,126 MAGs. After quality assessment (completeness ≥ 80% and contamination ≤ 5%) and dereplication at 99% average nucleotide identity (ANI), 7,530 non-redundant, high-quality MAGs were retained from the initial 10,594 MAGs and included in subsequent analyses (Supplementary Table 3). Additionally, this study constructed a gene catalog containing 14,840,909 genes, all of which contain complete open reading frames (ORFs) (Figure 1A). These 7,350 MAGs exhibited genome sizes ranging from 0.45 to 8.15 Mbp (average 2.19 Mbp) and GC contents ranging from 22.58% to 73.02% (average 46.70%) (Figure 1B). The average completeness of the MAGs was 89.73%, with average contamination was 1.35% (Figure 1C).

To classify the gut microbiota of Caprinae animals, taxonomic annotation revealed assignments to 28 phyla, 41 classes, 100 orders, 233 families, 1,032 genera and 2,486 species. The most dominant phylum was Bacillota_A (44.17%, *n* = 3,326), followed by Bacteroidota (*n* = 2,382). At the family level, the most prominent family include *Bacteroidaceae* (*n* = 837), *Lachnospiraceae* (*n* = 674), and *Acutalibacteraceae* (*n* = 615). At the genus level, *Prevotella* (*n* = 349), *Alistipes* (*n* = 338), and *Cryptobacteroides* (*n* = 255) were the most prominent (Figure 1D and Supplementary Table 3).

### 3.2 Role of MAGs in BAs metabolism in the intestines of Caprinae animals

**FIGURE 2:**
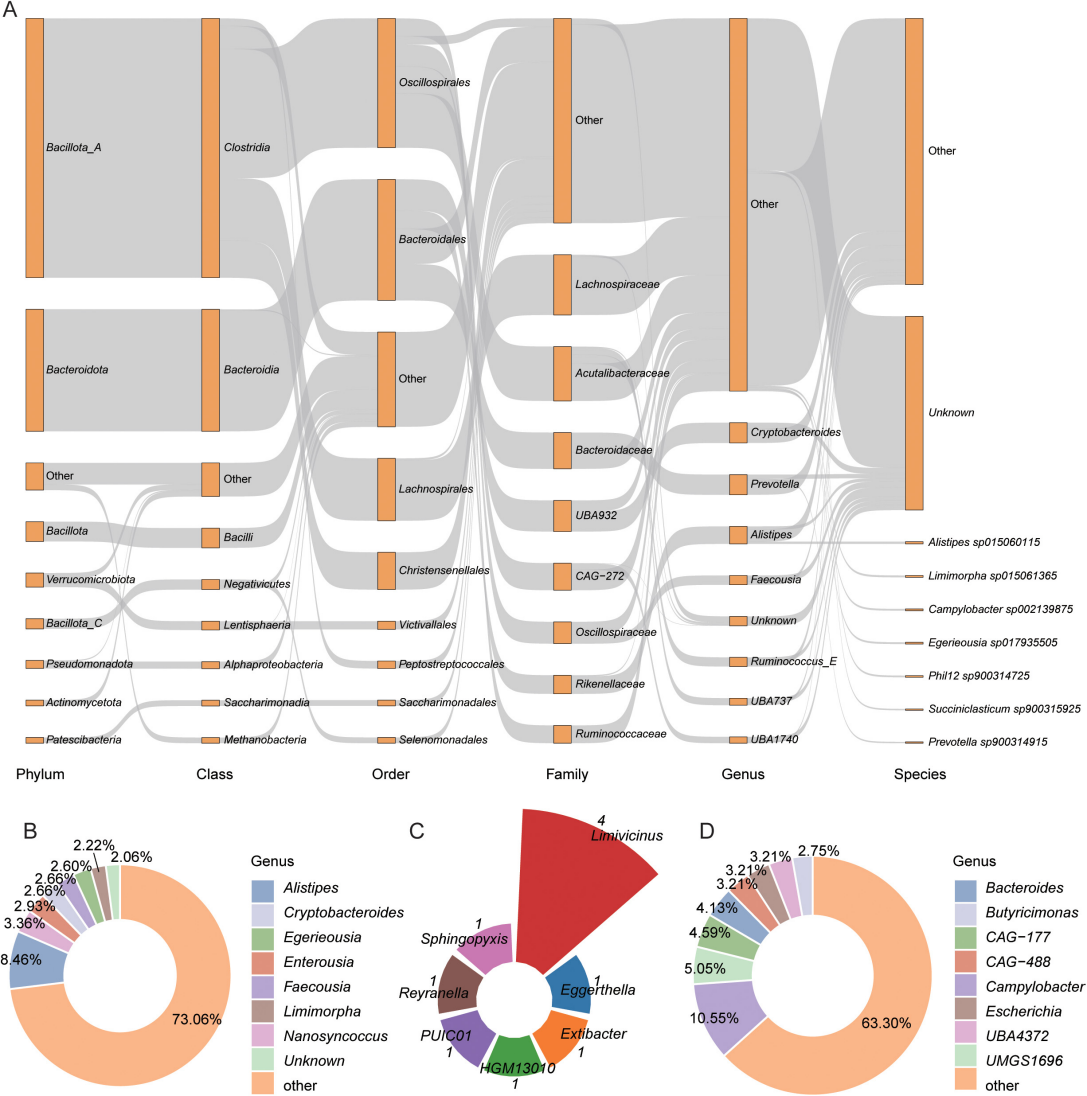
Overview of the 5,217 metagenome-assembled genomes (MAGs) identified to bile acids (BAs) biosynthesis. **(A)** Taxonomic classification of 5,217 BAs transformation MAGs across hierarchical levels. Rectangles represent different taxonomic ranks, with their lengths proportional to the number of genomes assigned to each level. **(B–D)** Proportions of genomes encoding key enzymes involved in BA metabolism: **(B)** bile salt hydrolase (BSH), **(C)** 7α-hydroxysteroid dehydrogenase (7α-HSDH), and **(D)** bile acid-inducible CoA ligase (baiB).

This study identified the BA biosynthesis pathways mediated by the gut microbiota in Caprinae animals through KEGG functional annotation. A total of 8,983,596 genes were annotation KOs. A total of 8,290 genes from 5,217 genomes were found to be involved in BA transformation pathways, including deconjugation, oxidation, and dehydroxylation (Supplementary Table 4). To gain insights into microbe-mediated BAs metabolism, this study performed a taxonomic analysis of BAs biosynthetic genes. Bacteria from the Bacillota_A phylum (primarily *Clostridia*) were the dominant carriers of BA-related KOs, followed by Bacteroidota and Bacillota. At the family level, *Lachnospiraceae* was the most abundant, followed by *Acutalibacteraceae* and *Bacteroidaceae* (Figure 2A).

Among the 5,217 MAGs, 1,845 MAGs encoded bile salt hydrolase (BSH) (choloylglycine hydrolase, K01442), an enzyme that hydrolyzes conjugated bile salts into deconjugated BAs. These MAGs were classified into 10 phyla, with the largest proportion from Bacillota_A (51.44%, *n* = 949), followed by Bacteroidota (29.92%, *n* = 552) (Supplementary Figure 1A). This indicates that multiple phylogenetically diverse microbial lineages in the intestines of Caprinae animals possess the capability for bile salt hydrolysis, underscoring the functional redundancy and ecological significance of this metabolic pathway within the ruminant gut. The majority of BSH-carrying genomes were assigned to the families *Acutalibacteraceae* (12.3%, *n* = 227), *Lachnospiraceae* (11%, *n* = 203), and CAG-272 (10.78%, *n* = 199) (Supplementary Figure 1B), with the genera *Alistipes* (8.46%, *n* = 156) and *Nanosyncoccus* (3.36%, *n* = 62) being the most predominant (Figure 2B).

In contrast, only 10 MAGs, from three phyla—Bacillota_A (88.24%), Pseudomonadota, and Actinomycetota (11.76%) (Supplementary Figure 1C)—encoded baiB gene (bile acid-coenzyme A ligase K15868), an enzyme that plays a crucial role in the bile acid transformation process. In genus levels, 4 MAGs were *Limivicinus* (Figure 2C). Additionally, 218 MAGs encoded 7α-HSDH (7-alpha-hydroxysteroid dehydrogenase, K00076), an enzyme that oxidizes the hydroxyl group of deconjugated BAs in an NAD(P) + -dependent manner. These MAGs were predominantly from Bacillota_A (49.54%, *n* = 108), followed by Bacteroidota (20.64%, *n* = 45) (Supplementary Figure 1D). In genus levels, *Campylobacter* were the most (Figure 2D).

### 3.3 Distinct BA metabolism in the intestines of Caprinae animals

**FIGURE 3:**
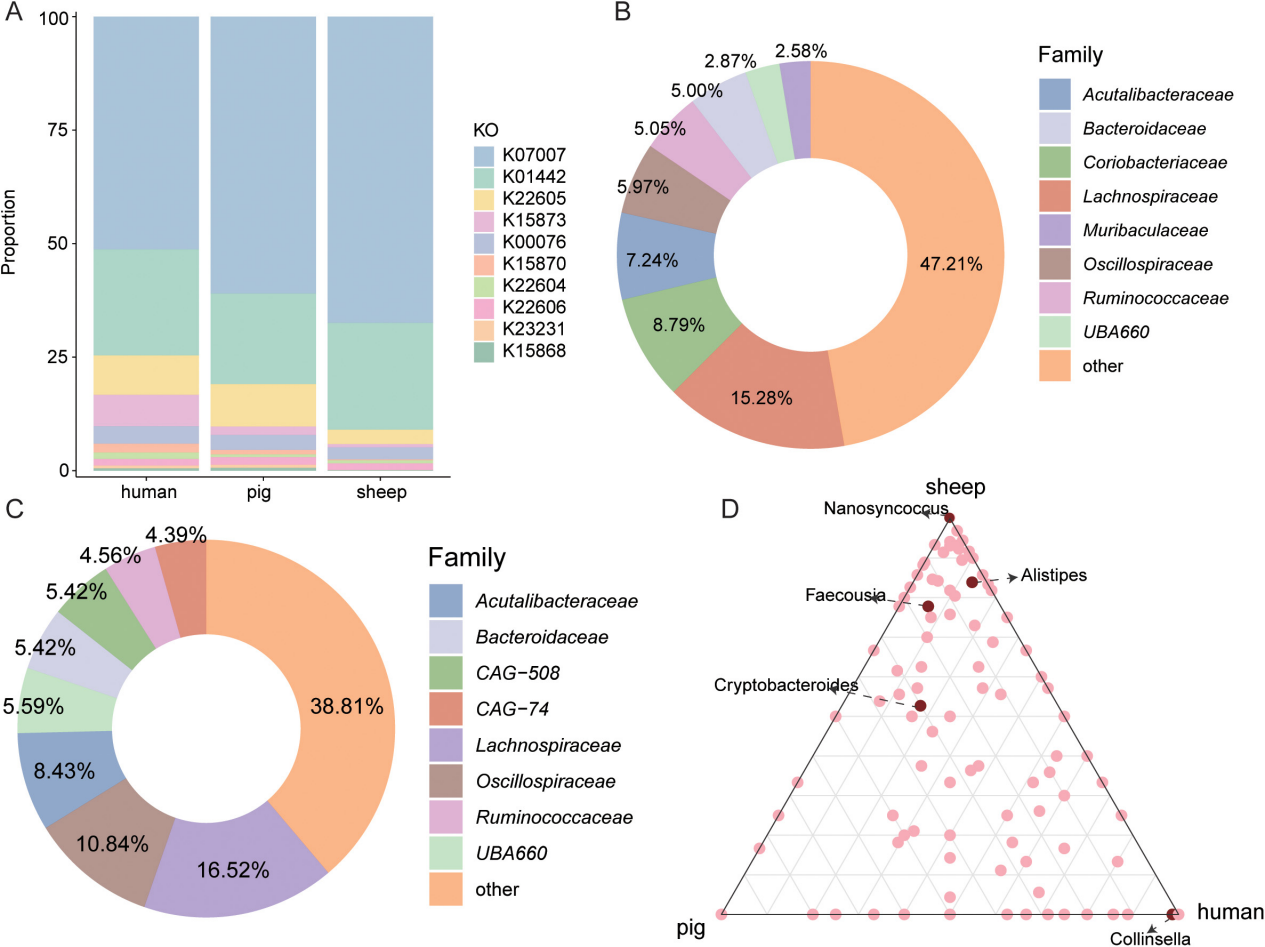
Distinct bile acids (BAs) metabolism in the Caprinae animal gut. **(A)** Stacked bar graphs depict the community composition of the BAs metabolism KEGG orthologs (KOs) of diff animals. **(B,C)** The proportion of BAs-metabolizing bacterial families in humans and pigs. **(D)** Classification of bile salt hydrolase (BSH)-carrying metagenome-assembled genomes (MAGs) at the genus level in the intestine of Caprinae animals, humans, and pigs.

The BA-metabolizing specificity of microorganisms in the intestines of Caprinae animals was investigated by comparing the 5,217 intestinal MAGs obtained in this study with those from other species: humans (2,294 MAGs) (Nayfach et al., 2019) and pigs (1,411 MAGs) (Gaio et al., 2021) which reported in previous studies. Taxonomic and functional annotation revealed 3,499 BA-related KOs in 2,294 human genomes and 2,229 KOs in 1,411 pig genomes (Supplementary Table 5). Compared with humans and pigs, Caprinae animals have the highest proportion of BSH-related genes and the lowest proportion of baiA-related (K22605) genes (Figure 3A). Among the intestinal microbiota, *Lachnospiraceae* represented one of the main bile acid-metabolizing bacterial families in humans (15.28%), pigs (16.52%), and sheep (12.77%). In addition, *Coriobacteriaceae* exhibited the highest relative abundance among BA-metabolizing taxa in humans, while its proportion was lower in pigs and Caprinae animals (Figures 3B, C).

Regarding BA deconjugation, BSH was widely distributed across host species, with 31.08% of human intestinal MAGs, 29.20% of pig MAGs, and 35.37% of Caprinae animals gut MAGs carrying BSH. *Nanosyncoccus* and *Alistipes* were the top BSH-carrying genus in the intestine of Caprinae animals but it accounted for a lower proportion of BSH-carrying MAGs in humans and pigs. Also, *Collinsella* was the top BSH-carrying genus in the intestine of human and it accounted for a lower proportion of BSH-carrying MAGs in pigs and Caprinae animals (Figure 3D).

### 3.4 Function potential of BSH-carrying MAGs of the intestines of Caprinae animals

The initial “gateway reaction” in bile acid (BA) metabolism is the deconjugation of primary BAs, a process catalyzed by bile salt hydrolase (BSH). This reaction plays a pivotal role in microbial bile tolerance and adaptation to the selective pressures of the intestinal environment (Guzior and Quinn, 2021; Jones et al., 2008). To investigate the functional potential of BSH-encoding metagenome-assembled genomes (MAGs) in the intestinal microbiota of Caprinae animals, this study performed a comparative genomic analysis within the genus *Alistipes*.

**FIGURE 4:**
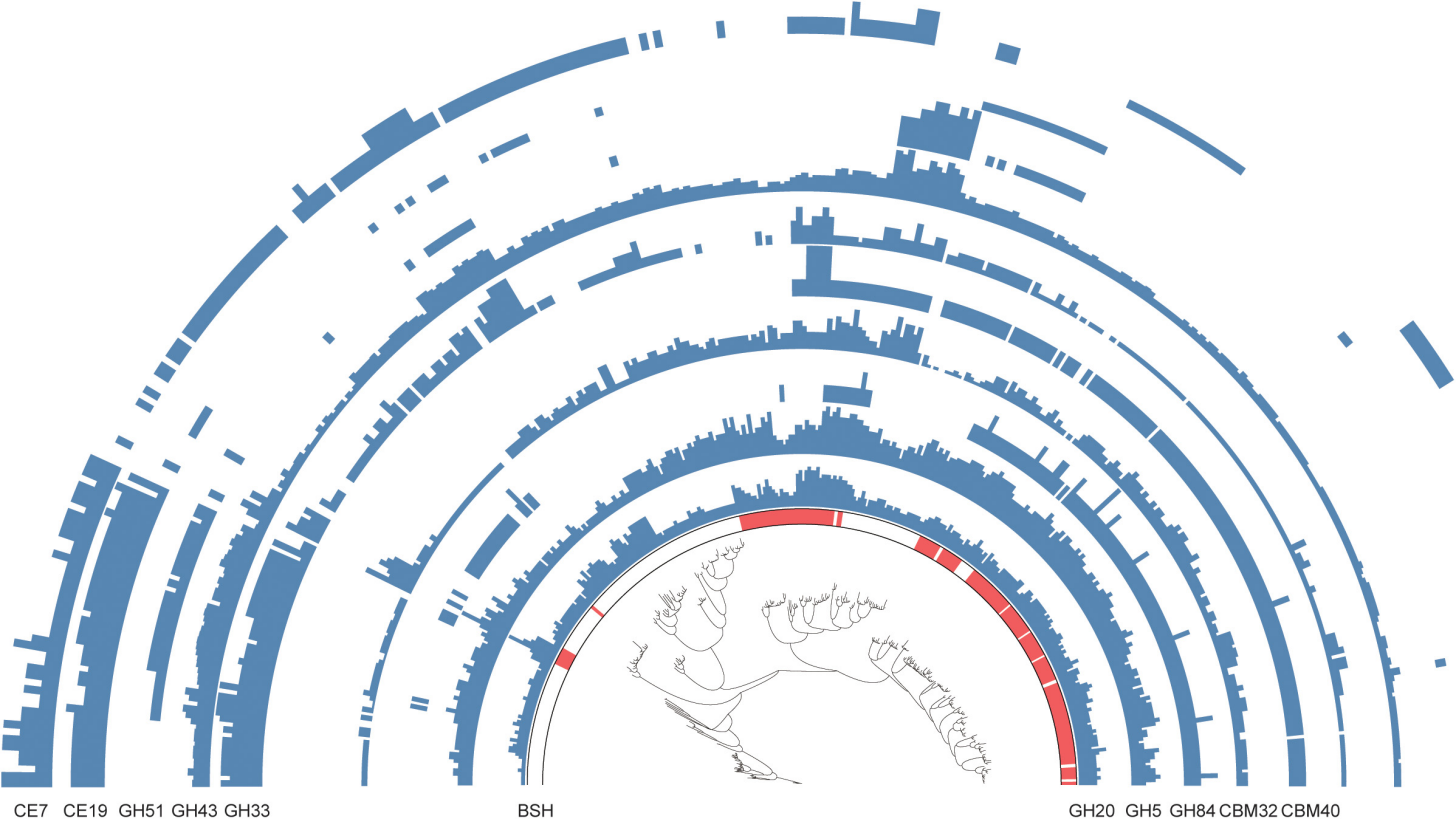
Functional advantages of bile salt hydrolase (BSH)-carrying metagenome-assembled genomes (MAGs) in the intestine of Caprinae animals. Comparison of the functional differences in carbohydrate-active enzymes between BSH-carrying and non-BSH-carrying MAGs belonging to *Alistipes*.

Among the 338 *Alistipes* MAGs identified, 182 encoded BSH genes, while the remaining 156 were classified as non-BSH genomes (Supplementary Table 6). Functional annotation based on carbohydrate-active enzymes (CAZymes) revealed marked differences between BSH-carrying and non-BSH-carrying genomes. Notably, MAGs encoding BSH showed a significantly higher prevalence of CAZyme families such as CBM40, GH5, GH84, and GH20, which are closely associated with complex carbohydrate degradation and microbial adaptation to the gut niche. An intriguing observation was the exclusive presence of certain CAZyme families-such as CBM40 and GH84-in BSH-carrying genomes, which were entirely absent from non-BSH-carrying MAGs. Conversely, CAZymes including CE19 and GH51 were detected exclusively in non-BSH-encoding genomes and were completely absent from those carrying BSH (Figure 4 and Supplementary Table 6). These findings suggest that BSH-positive and BSH-negative *Alistipes* populations possess distinct metabolic repertoires, potentially reflecting divergent ecological roles in the intestinal environment.

### 3.5 Modification of the BAs metabolism signature among different intestinal segments of *Ovis aries*

**FIGURE 5:**
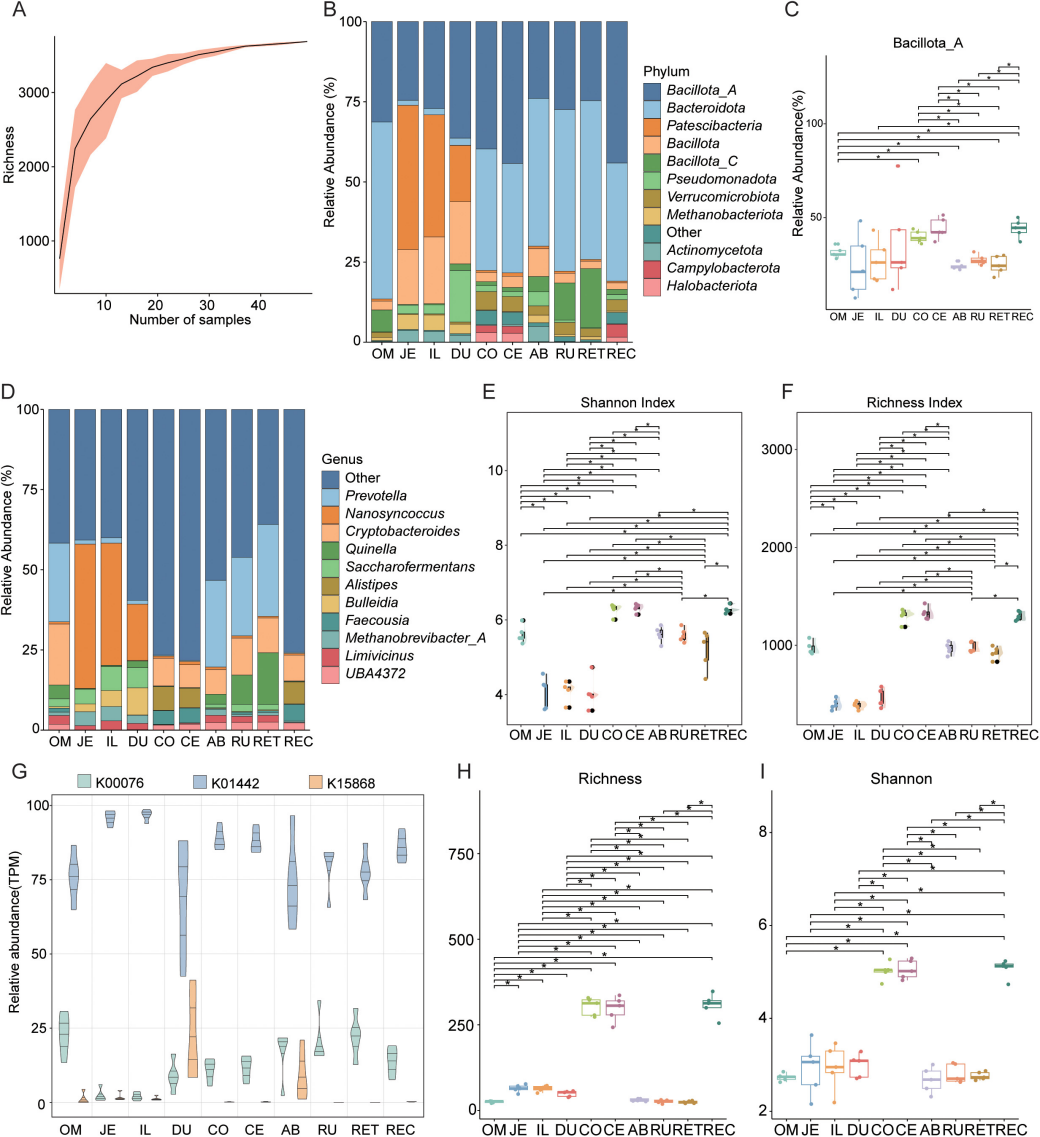
Analysis of differences in bile acids (BAs) metabolic microorganisms and bile salt hydrolase (BSH) across different intestinal sites. **(A)** Rarefaction curve analysis showing bacterial species richness. **(B)** Stacked bar graphs depict the community composition of the microbiota at phylum levels. **(C)** Boxplots showing the relative abundance of Bacillota_A across ten different intestinal sites. **(D)** Stacked bar graphs depict the community composition of the microbiota at genus levels. **(E,F)** Boxplots illustrating the species Shannon and Richness indices across ten different intestinal sites, respectively. **(G)** Relative abundance of three BAs metabolism-related enzyme genes across ten different intestinal sites. **(H,I)** Boxplots illustrating the species Richness and Shannon indices across ten different intestinal sites, respectively. Significance levels were determined using the Wilcoxon rank-sum test: *, *p* < 0.05. RU, rumen; RET, reticulum; REC, rectum; OM, omasum; JE, jejunum; IL, ileum; DU, duodenum; CO, colon; CE, cecum; AB, abomasum.

To systematically characterize the BAs-metabolizing potential across the 10 intestinal segments of *Ovis aries*, taxonomic composition and BA profiles were compared. Rarefaction curve analysis showed that as the number of sequencing samples increased, taxonomic richness gradually approached a plateau, indicating that the sample size in this study was sufficient to capture the microbial diversity associated with BA metabolism in different intestinal regions of *Ovis aries* (Figure 5A).

At the phylum level, Bacillota_A and Bacteroidota were the dominant taxa with the highest relative abundances (Figure 5B). Notably, the abundance of Bacillota_A in the abomasum (AB) was significantly lower than that in the omasum (OM), colon (CO), cecum (CE), and rectum (REC) (*p* < 0.05, Wilcoxon rank-sum test; Figure 5C). At the genus level, microbial composition varied among the ten intestinal segments of *Ovis aries* (Figure 5D). Specifically, the relative abundance of *Prevotella* was significantly higher in the OM, AB, rumen (RU), and reticulum (RET) compared to other segments (*p* < 0.05, Wilcoxon rank-sum test; Supplementary Figure 2A). The genus *Nanosyncoccus* showed the highest relative abundance among all intestinal regions and was significantly more abundant than in the CO, CE, AB, RU, RET, and REC (*p* < 0.05; Supplementary Figure 2B). In addition, *Cryptobacteroides* was significantly more abundant in the OM than in other segments (*p* < 0.05, Wilcoxon rank-sum test; Supplementary Figure 2C). Similarly, *Quinella* displayed higher relative abundance in the OM compared to the jejunum (JE), CO, and CE (*p* < 0.05; Supplementary Figure 2D). PCoA revealed clear separation between intestinal segments (*p* = 0.001; Supplementary Figure 2E).

In this study, the diversity of microbial communities across different intestinal segments was assessed. Alpha-diversity analysis based on the Shannon and Richness indices showed that the microbial community diversity in the colon (CO), cecum (CE), and rectum (REC) was significantly higher than in other intestinal segments (*p* < 0.05, Wilcoxon rank-sum test; Figures 5E, F). Further analysis revealed a widespread presence of key BAs biosynthesis enzymes-bile salt hydrolase (BSH), 7α-hydroxysteroid dehydrogenase (7α-HSDH), and bile acid-inducible CoA ligase (baiB)-across all ten intestinal segments of *Ovis aries* (Figure 5G). Notably, the jejunum (JE) and ileum (IL) exhibited higher abundances of these enzymes compared to other intestinal segments. These findings suggest that specific intestinal regions influence gut microbial composition and BA biosynthesis potential. To further examine the distribution of BSH-producing microorganisms, α-diversity analysis based on the Shannon and Richness indices again demonstrated that microbial diversity in the CO, CE, and REC was significantly higher than in other segments (*p* < 0.05, Wilcoxon rank-sum test; Figures 5H, I).

## 4 Discussion

The study provides a comprehensive analysis of the gut microbiota in Caprinae animals, focusing on bile acid (BA) metabolism. In terms of BA metabolism, 8,290 genes from 5,217 genomes were identified as involved in BA transformation pathways, including deconjugation, oxidation, and dehydroxylation. *Alistipes* and *Nanosyncoccus* were the most prominent carriers of bile salt hydrolase (BSH) genes, while *Limivicinus* was notable for carrying the baiB gene. Comparative analysis with human and pig gut microbiota revealed that Caprinae animals harbor a higher proportion of BSH-related genes and a lower proportion of baiA-related genes, indicating distinct BA metabolic profiles across difference in host physiology and diet (Collins et al., 2023). Additionally, the study found functional advantages in BSH-carrying MAGs, such as higher prevalence of CAZymes associated with gut adaptation. Microbial diversity and BA metabolism potential also varied across different intestinal segments of Caprinae animals, with the colon, cecum, and rectum exhibiting higher microbial diversity, while the jejunum and ileum had higher abundances of BA metabolism-related enzymes.

The high diversity of the Caprinae gut microbiota, dominated by Bacillota_A and Bacteroidota, aligns with previous studies on ruminants (Luo et al., 2022). The prevalence of *Clostridia* within Bacillota_A is particularly noteworthy, as these bacteria play crucial roles in BA metabolism. Their widespread presence suggests functional specialization and ecological adaptation within the Caprinae intestinal tract. These adaptations may reflect long-term co-evolution and symbiotic interactions that promote both microbial persistence and host physiological benefits, particularly in lipid digestion and immune modulation (Cui et al., 2023; Wang et al., 2023). The identified BA-metabolizing taxa may serve as candidates for probiotic development aimed at improving gastrointestinal health in veterinary and wildlife settings (O’Toole et al., 2017).

Bile tolerance is a crucial trait of microbial consortia, as it determines the ability of a strain to survive in the intestine (Winston and Theriot, 2020). In this study, the bacteria involved in intestinal bile acid metabolism in sheep subfamily animals, humans, and pigs primarily belonged to the *Lachnospiraceae* family, whereas previous studies on dairy cows identified the *Acutalibacteraceae* family as the dominant group (Lin et al., 2023; Hofmann et al., 2010; Wang and Carey, 2014). This highlights the interspecies differences in gut microbial composition and bile acid metabolism. BSH activity allows microbes to hydrolyze conjugated bile salts, which is essential for microbial survival in the bile-rich gut environment and also impacts host lipid metabolism (Yang and Wu, 2022). From a host-specific perspective, our study revealed a higher prevalence of bile salt–hydrolyzing capacity in *Alistipes* populations from Caprinae than in those from humans or pigs, which may be linked to the distinctive rumen–intestine physiology of ruminants. This observation is in line with previous findings on bile acid metabolism by intestinal microorganisms in dairy cows (Lin et al., 2023), suggesting that *Alistipes* may play a conserved role in BA activity within ruminant hosts. In contrast, the lower abundance of baiB genes suggests that Caprinae gut microbiota may have a distinct role in BA synthesis compared to other animals like humans and pigs (Collins et al., 2023). To further strengthen these conclusions, future studies incorporating additional ruminant species, such as dairy and beef cattle, will be valuable. Such comparative analyses may refine our understanding of host-specific bile acid metabolic pathways and support the identification of functional traits unique to Caprinae gut microbiota.

*Alistipes*, a genus known for metabolizing host-derived compounds, may contribute to diversification of the BA pool and related signaling pathways, extending the functional repertoire beyond well-studied genera like *Bacteroides* (Parker et al., 2020). These taxonomic associations expand our understanding of BA-metabolizing lineages beyond classical human-associated genera such as Bacteroides, offering new candidates for targeted functional validation. The functional advantages of BSH-carrying MAGs, such as higher prevalence of CAZymes like CBM40 and GH5, indicate that these microbes are better adapted to the gut environment (Lee et al., 2024). This adaptation likely enhances their ability to degrade complex carbohydrates and compete for resources within the gut (Flint et al., 2012). Such functional traits may have evolved through co-evolution with the host, promoting a symbiotic relationship that benefits both the microbes and the host’s metabolic health (Collins et al., 2023).

The variation in microbial diversity and BA metabolism potential across different intestinal segments of Caprinae animals highlights the influence of local gut conditions on microbial community structure (Lv et al., 2023). Consistent with previous findings in ruminants, the colon, cecum, and rectum harbor higher microbial diversity and taxonomic richness, likely because these distal gut regions provide an anaerobic environment and nutrient-rich conditions that support complex fermentative communities (Seshadri et al., 2018; Lin et al., 2023). In contrast, the enrichment of BA metabolism-related enzymes in the jejunum and ileum suggests that these proximal regions play a pivotal role in bile acid processing and absorption (Devkota et al., 2012). This spatial separation of microbial functions reflects a clear division of labor along the gastrointestinal tract, whereby the proximal segments optimize nutrient absorption and BA transformation, while the distal segments maintain fermentative capacity and microbial diversity. Such compartmentalization likely promotes host–microbe co-adaptation, ensuring both metabolic efficiency and BA homeostasis.

## 5 Conclusion

This study provides a comprehensive genome-resolved overview of bile acid (BA)-metabolizing microbiota in the gastrointestinal tract of Caprinae animals. By analyzing 6,332 high-quality genomes across ten intestinal regions, we uncovered distinct microbial communities and spatially specialized patterns of BA transformation. The jejunum and ileum harbored the highest abundances of key BA-metabolizing enzymes, while the colon, cecum, and rectum displayed greater microbial diversity. Taxa such as *Alistipes* and *Nanosyncoccus* emerged as dominant contributors to BA metabolism, with functional enrichment in both BA deconjugation and carbohydrate utilization. These findings underscore the compartmentalized and host-adapted nature of microbial BA metabolism in ruminants, offering a valuable framework for future studies on microbiota-mediated modulation of host metabolism and gut health in livestock.

## Data Availability

The original contributions presented in this study are included in this article/Supplementary material, further inquiries can be directed to the corresponding authors.

## Supplementary material

Supplementary Figure 1 The proportion of metagenome-assembled genomes (MAGs) encoded bile salt hydrolase (BSH), 7α-hydroxysteroid dehydrogenase (7α-HSDH), and bile acid-inducible CoA ligase (baiB). **(A)** Proportions of genomes encoding BSH in phylum. **(B)** Proportions of genomes encoding BSH in family. **(C)** Proportions of genomes encoding baiB in family. **(D)** Proportions of genomes encoding BSH in phylum.

Supplementary Figure 2 Analysis of differences in bile acid (BA) metabolic microorganisms across different intestinal segments. **(A–D)** Boxplots showing the relative abundance of *Prevotella*, *Nanosyncoccus*, *Cryptobacteroides* and *Quinella* across ten different intestinal sites. Significance levels were determined using the Wilcoxon rank-sum test: *, *p* < 0.05; **, *p* < 0.01, ***, *p* < 0.001. **(E)** The scatter plot illustrates the β-diversity of genes associated with BA metabolism across various intestinal segments. Samples are projected onto the first two principal coordinate axes (PCoA1 and PCoA2), with the percentage of variance explained by each axis indicated. Kernel density estimation curves for PCoA1 and PCoA2 are displayed along the top and right margins, respectively. Permutational multivariate analysis of variance (PERMANOVA) results are shown in the bottom right corner, where point color reflects the *p*-value magnitude, and point size corresponds to the R^2^ value, indicating the proportion of variance explained. RU, rumen; RET, reticulum; REC, rectum; OM, omasum; JE, jejunum; IL, ileum; DU, duodenum; CO, colon; CE, cecum; AB, abomasum.
