# Supplementary figures and images for: Bile acid pathways in Caprinae gut microbiota: adaptive shifts in microbial metabolism and community structure
Source: Front Microbiol. 2025 Sep 12;16:1648896. doi: 10.3389/fmicb.2025.1648896 (PMC12466152; doi:10.3389/fmicb.2025.1648896)

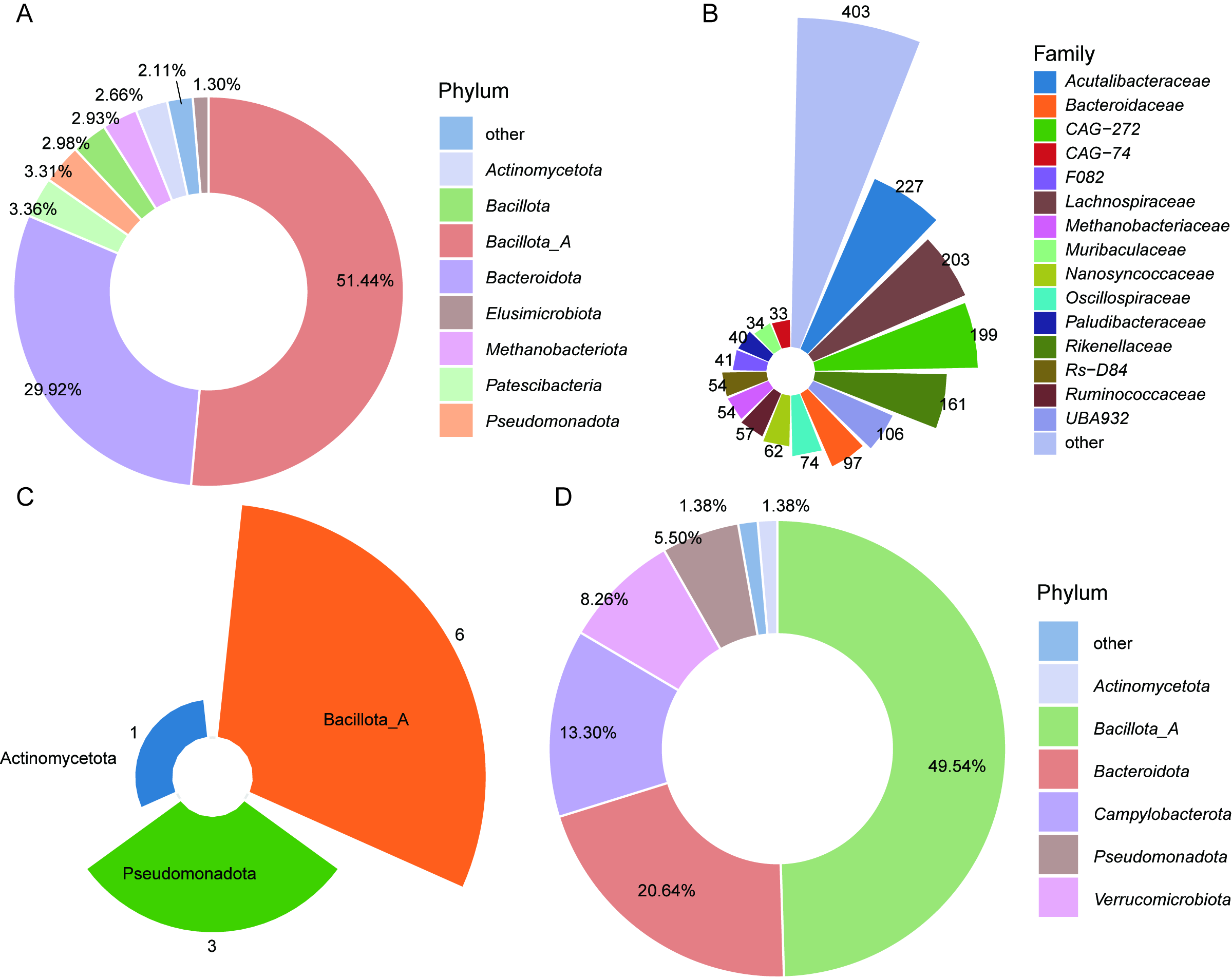

Supplement: Supplementary Figure 1 — The proportion of metagenome-assembled genomes (MAGs) encoded bile salt hydrolase (BSH), 7α-hydroxysteroid dehydrogenase (7α-HSDH), and bile acid-inducible CoA ligase (baiB). (A) Proportions of genomes encoding BSH in phylum. (B) Proportions of genomes encoding BSH in family. (C) Proportions of genomes encoding baiB in family. (D) Proportions of genomes encoding BSH in phylum. [file Image_1.tif]

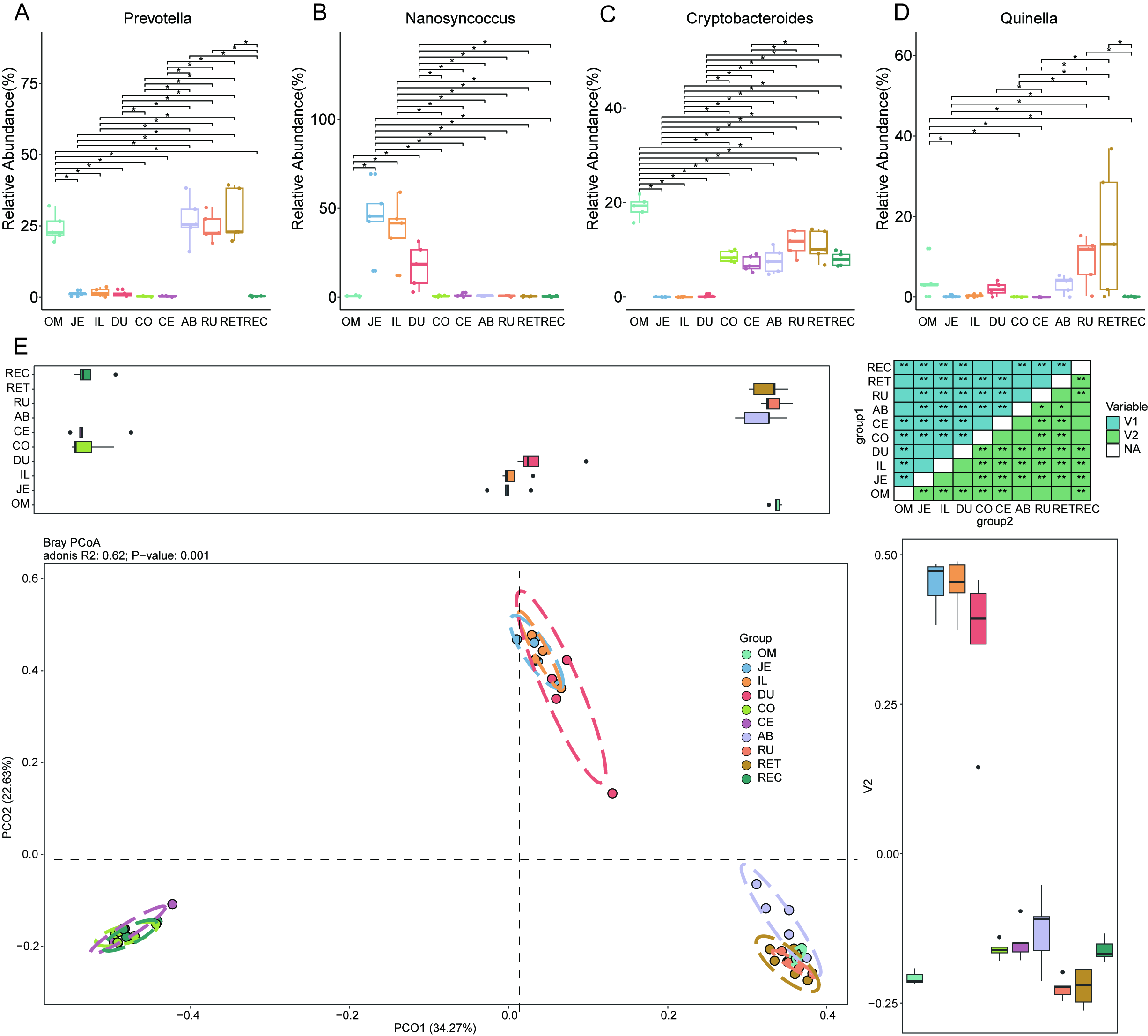

Supplement: Supplementary Figure 2 — Analysis of differences in bile acid (BA) metabolic microorganisms across different intestinal segments. (A–D) Boxplots showing the relative abundance of Prevotella, Nanosyncoccus, Cryptobacteroides and Quinella across ten different intestinal sites. Significance levels were determined using the Wilcoxon rank-sum test: *, p < 0.05; **, p < 0.01, ***, p < 0.001. (E) The scatter plot illustrates the β-diversity of genes associated with BA metabolism across various intestinal segments. Samples are projected onto the first two principal coordinate axes (PCoA1 and PCoA2), with the percentage of variance explained by each axis indicated. Kernel density estimation curves for PCoA1 and PCoA2 are displayed along the top and right margins, respectively. Permutational multivariate analysis of variance (PERMANOVA) results are shown in the bottom right corner, where point color reflects the p-value magnitude, and point size corresponds to the R2 value, indicating the proportion of variance explained. RU, rumen; RET, reticulum; REC, rectum; OM, omasum; JE, jejunum; IL, ileum; DU, duodenum; CO, colon; CE, cecum; AB, abomasum. [file Image_2.tif]
